# Improving the Quality of Provided Care: Lessons Learned From Auditing Neonatal Stabilization

**DOI:** 10.3389/fped.2020.00560

**Published:** 2020-09-16

**Authors:** Maria C. den Boer, Tessa Martherus, Mirjam Houtlosser, Laura Root, Ruben S. G. M. Witlox, Arjan B. te Pas

**Affiliations:** ^1^Division of Neonatology, Leiden University Medical Center, Leiden, Netherlands; ^2^Department of Medical Ethics and Health Law, Leiden University Medical Center, Leiden, Netherlands

**Keywords:** neonatal stabilization, audit and feedback, quality improvement, video recording, patient safety

## Abstract

Video and physiological parameter recording of neonatal stabilization was implemented at the Neonatal Intensive Care Unit (NICU) of the Leiden University Medical Center. In order to improve documentation and the quality of care provided during neonatal transition, we implemented weekly plenary audits reviewing recordings of neonatal stabilization in 2014. In audits, provided care is reviewed, discussing, among others, mask technique, compliance to the prevailing local guideline, and clinical decision making and alternative treatment options. In this perspective, we argue that auditing neonatal stabilization is a valuable tool to improve patient safety and the quality of care provided during neonatal stabilization. We, therefore, report lessons learned and areas for improvement that could be identified and addressed during audits conducted at our NICU. Important areas for improvement were guideline compliance, documentation, the usage of medical devices, the conduct of delivery room studies, and clinical decision making. By reporting our experiences, we hope to encourage other NICUs to also implement regular audit meetings, fitting to their improvement needs.

## Introduction

**Figure 1:**
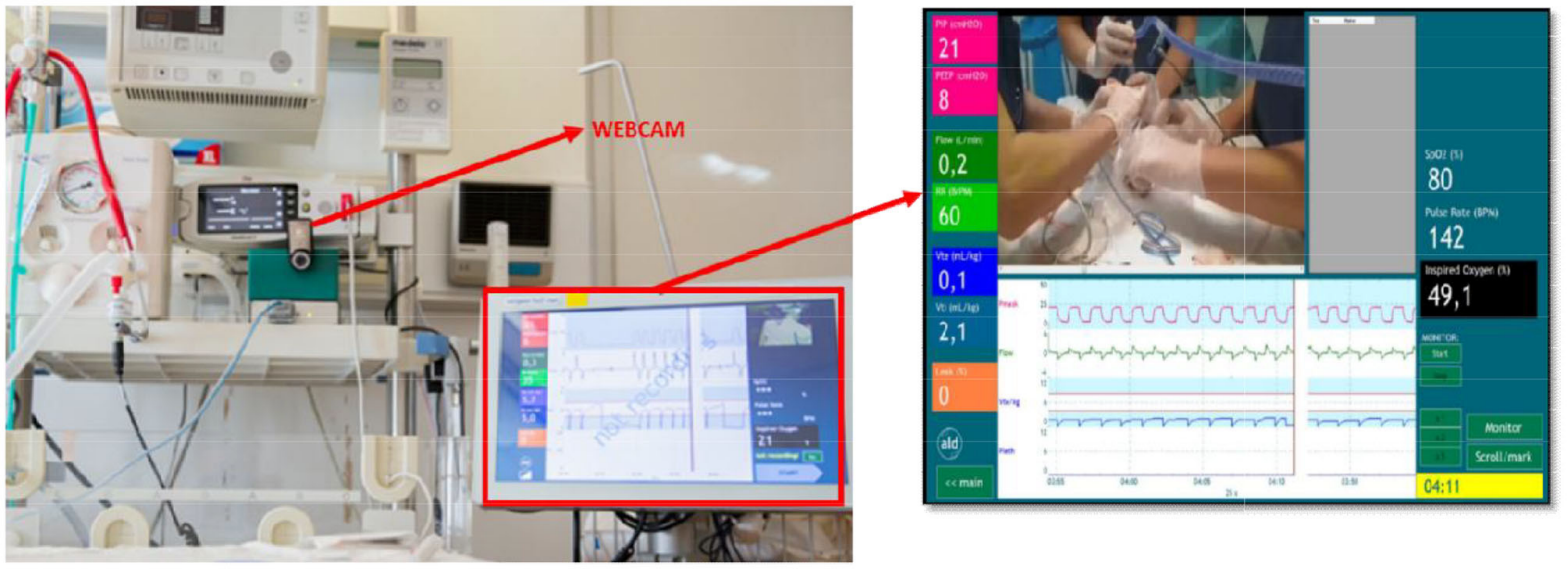
Recording equipment. Video is recorded by a webcam, capturing the neonate, and providers' hands. Vital parameters include positive end-expiratory pressure and peak inspiratory pressure (pink), flow and respiratory rate (green), expired tidal volume (blue) and mask leak (orange), oxygenation, heart rate, and fraction of inspired oxygen. All measurements are digitized and recorded at 200 Hz using the Bicore II (Cardinal Healthcare, Yorba Linda, CA, USA) physiological recording system with Polybench software (Advanced Life Diagnostics, Weener, Germany).

Providing support to neonates during neonatal transition is challenging. In order to study care provided during neonatal transition, we implemented recording of video and physiological parameters of neonatal stabilization at the Neonatal Intensive Care Unit (NICU) of the Leiden University Medical Center (LUMC), a tertiary-level perinatal center with an average of 850 admissions a year, in 2009. In order to protect the privacy of providers, recordings capture only imaging of the neonate and providers' hands (Figure 1). Evaluation of recordings of neonatal stabilization showed that providers frequently diverge from the prevailing guidelines and that mask technique is often inadequate (1). Comparing recordings with the medical record furthermore showed that documentation in the medical record is often incomplete or inaccurate (2). In order to improve documentation and the quality of care provided during neonatal transition, we implemented weekly plenary audits reviewing recordings of neonatal stabilization in 2014.

Our weekly audits are prepared and chaired by the coordinator of the audit. Providers can request a specific stabilization to be discussed, or the coordinator selects a stabilization based on gestational age (<26 weeks), intensity of provided care (e.g., intubation, cardiac resuscitation), medical history (e.g., congenital malformation, twin–twin transfusionm syndrome), or inclusion in a delivery room study. Audits take place after morning handover and last ~20 min. All NICU staff members are invited to participate in these meetings. In audits, provided care is reviewed, discussing, among others, mask technique, compliance to the prevailing local guideline, and clinical decision making and alternative treatment options. By concluding the audit, providers capture lessons learned. Since 2018, minutes are sent out after every audit, as this allows providers that could not have been present during the specific audit to also learn from the discussed stabilization.

Various studies highlight the clear benefits of recording and auditing actual care (3, 4), yet several studies failed to prove improvements in clinical performance after auditing recordings of actual neonatal stabilization (5–7). Recently, we reported that weekly plenary audits at our NICU improved guideline compliance and documentation in the medical records (8). Furthermore, we reported that providers report various educational benefits of auditing recordings of neonatal stabilization and that providers consider audits beneficial for improving the quality of care provided during neonatal transition. Providers, therefore, not only recommend the implementation of plenary audits but also acknowledge that successful implementation requires that audits should complement the needs of a NICU (9). Based on experiences at our center, we argue that auditing neonatal stabilization is a valuable tool in improving patient safety and the quality of care provided during neonatal stabilization, as it can be used to identify and address various areas for improvement. By providing insight in these areas for improvement, we hope to encourage other NICUs to implement regular plenary audits using recordings of neonatal stabilization, fitting to their specific needs.

## Lessons Learned During Plenary Audits

In order to provide more insight in the potential of plenary audits in improving the quality of provided care during neonatal stabilization, we analyzed all notes made during audits and minutes sent out after audits in the period between February 2018 and February 2019. In this period, 39 stabilizations were discussed during plenary audit meetings. Infants that received support during these stabilizations were born after a median (range) gestational age of 27 + 4 (24 + 3–41 + 5) weeks. In total, 131 lessons learned were captured, with median (IQR), 3 (2–4) lessons learned per audit. These lessons learned were connected to 15 areas for improvement.

Audits were attended by a mean ± SD of 23 ± 4 staff members. This group was averagely composed of 5 ± 2 consultants, 3 ± 1 fellows, 4 ± 1 residents, 2 ± 1 physician assistants, 1 ± 1 nurse, and 7 ± 1 staff members not involved in hands-on care (e.g., coordinator of the audit, investigators, and medical students). During 12 (31%) audits, members of the obstetrical team attended the audit after the joint handover of the NICU and obstetrics department.

Of all 131 captured lessons learned, 50 (38%) were beneficial for the medical team and 4 (3%) for the nursing team. Another 53 (40%) lessons learned were beneficial for both the medical and the nursing team. Of the remaining 24 (18%) lessons learned, 11 (8%) were beneficial for obstetrics and 13 (10%) for staff members not involved in hands-on care. The lessons learned revealed various areas for improvement. Table 1 provides an overview of the lessons learned and connected areas for improvement. We will shortly discuss the most important lessons learned.

**Table 1:** Lessons learned during plenary audits.

**Lesson learned during plenary audits (*****n*** **=** **131)**			
		***n*** (%)^*^	**Examples**
Discipline	Medical staff	50 (38)	Standard operating procedures of delivery room studies, oxygen titration
	Nursing staff	4 (3)	Documentation, stimulation
	Both	53 (40)	Order of starting up devices, interpretation of monitor signals, dedicated persons
	Obstetrics staff	11 (8)	Interprofessional communication
	Not involved in hands-on care	13 (10)	Feedback on audit or study equipment
Skills	Technical	103 (79)	Medical devices, mask technique
	Non-technical	23 (18)	Communication, role differentiation
	Both	5 (4)	Role differentiation based on the physiology of delayed cord clamping
Area of improvement	Medical devices	25 (19)	Order of starting up of devices, placement of sensors
	Conduct of delivery room studies	24 (18)	Study protocol adherence, equipment for data collection, standard operating procedures
	Clinical decision making	22 (14)	Oxygen titration, moment of line placement, or drug administration
	Physiology	15 (9)	Larynx function, breathing patterns, effect of maternal drug usage
	Mask technique	11 (7)	Balance mask leak vs. applying too much pressure
	Protocol establishment	9 (6)	Line placement, heat management during delayed cord clamping
	Documentation	9 (6)	Documentation of rectal temperature or time until cord clamping
	Role differentiation	8 (5)	Dedicated person for specific tasks, delegation of tasks
	Communication	7 (4)	Interprofessional communication
	Self-assurance	7 (4)	Self-assurance, peer support
	Conduct of audit	6 (4)	Providers informed, involved, and prepared for audit
	RFM	5 (3)	Interpretation of monitor signals
	Need for research	4 (3)	Neopuff^TM^ and CO_2_ retention, trigeminal reflex
	Heat management	4 (3)	Heat management during delayed cord clamping
	Hand hygiene	3 (2)	Hand hygiene using mobile phones

* *As lessons learned can be connected to different areas of improvement, n/% exceeds 131/100%. RFM, respiratory functioning monitor*.

### Captured Lessons Learned

In total, 108 (82%) lessons learned were connected to technical skills, of which 24 (22%) concerned medical devices. These included the preparation of equipment, e.g., the correct order of starting up devices, as well as the correct usage of devices, e.g., the correct placement of sensors. Another 23 (21%) lessons learned were connected to the conduct of delivery room studies, especially standard operating procedures, study protocol adherence, preventing missing values, and study equipment.

Nineteen (18%) of the lessons learned were connected to clinical decision making and alternative treatment options, e.g., oxygen titration, moment of drug administration, or line placement. Audits frequently resulted in plenary discussions in which providers tended to reach consensus on the best practice. Three times (8%) these discussions resulted in agreement on adaptation of the local guideline. These adaptations included initial evaluation before starting respiratory support, heat management during delayed cord clamping, and the option to perform line placement in the delivery room, instead of at the NICU, with a maximum of two attempts of line placements in the delivery room.

In 11 (28%) audits, providers transferred knowledge about physiology, resulting in 12 (11%) lessons learned. This included the physiology of transition, the physiology of breathing patterns, and the physiology of maternal drug usage. Eleven (10%) lessons learned were connected to mask technique, especially concerning leak during the provision of respiratory support. Another nine (10%) lessons learned were connected to documentation, five (5%) to the respiratory functioning monitor (RFM), four (4%) to heat management, and three (3%) to hand hygiene.

When lessons learned were connected to cognitive, social, or personal skills, they were categorized as non-technical skills. In total, 28 (21%) lessons learned were connected to non-technical skills. Of these, eight (29%) were connected to role differentiation and seven (25%) to (interprofessional) communication. Six times, NICU providers requested to review a specific stabilization during an audit for self-assurance. Doing so enabled providers to ask for feedback and peer support.

Plenary audits, furthermore, resulted in insight in gaps in scientific knowledge. Providers reported the need for further research on the impact of the Neopuff^TM^ on CO_2_ retention, delivery room management for infants with twin–twin transfusion syndrome, and the impact of the trigeminal reflex during neonatal stabilization. The latter was followed up upon by studying the effect of applying a face mask for respiratory support on breathing in preterm infants at birth (10).

## Discussion

When plenary audits were implemented at our NICU in January 2014, we aimed to improve guideline compliance and documentation in the medical record. Recently, we reported that plenary audits indeed improved documentation in the medical record and guideline compliance (8). During 1 year of plenary audits at our NICU, many other areas of improvement were identified and addressed, proving that plenary audits may not only improve guideline compliance and documentation but also can contribute to other improvements.

An important identified area for improvement was the usage of medical devices: of all 131 captured lessons learned, 25 (19%) involved medical devices. Guidelines such as from the International Liaison Committee on Resuscitation (11) and the European Resuscitation Council (11, 12) advise on the usage of various devices to maintain normothermia, to objectively assess the infant, and to provide respiratory support during neonatal stabilization (13). Preparation of equipment is one of the keys to successful neonatal stabilization (13). In audits, providers were frequently reminded about the correct order of starting-up devices, ensuring successful preparation of equipment needed for neonatal stabilization. Furthermore, providers were reminded about the correct usage of medical devices. Inappropriate use of devices commonly occurs and is reported to be associated with patient safety incidents, causing harm (14) or even death (15). Audits may, thus, help to ensure successful equipment preparation and to prevent inappropriate usage of devices.

Another important area for improvement was the conduct of delivery room studies: 24 (18%) lessons learned were connected to study protocols, especially study protocol adherence, data loss situations, and study equipment. At our NICU, one up to three interventional studies are continuously being conducted in the delivery room. In audits, providers often identified protocol deviations. Protocol deviations commonly occur in research, which can be problematic, as protocol deviations may result in patient harm (16) or an invalid research (17). Protocol deviations cannot be entirely avoided, but their occurrence and impact on patient safety and validity of research can be reduced (18). Audits help to identify and address protocol deviations. Consequently, providers may, for instance, be retrained in study procedures through simulation scenarios. Providing insight in non-compliance to study protocols may furthermore provide investigators with valuable insights, for instance, on the feasibility of an experimental intervention. This may, consequently, improve future delivery room study protocols and future care.

Twenty-two (14%) lessons learned were connected to clinical decision making and alternative treatment options. Clinical guidelines are established in order to standardize and clarify care, and improve efficiency, productivity, and safety (19). As providers are recommended to follow guidelines in order to improve neonatal stabilization and outcome (1), we aimed to improve guideline compliance through auditing. However, when discussing guideline compliance during audits, it also turned out that more clarity in the guideline is needed in order to guide clinical decision making. Moreover, providers agreed that the local guideline for neonatal stabilization could be improved upon. Furthermore, guidelines for neonatal stabilization are designed for a standardized patient (20), whereas the clinical practice of providing care to transitioning infants is often more unruly. In audits, providers frequently openly discussed delivery room management for infants in these non-standard situations and the appropriateness of interventions for non-standard infants, trying to reach consensus on the best care, thus, harmonizing care. Moreover, as well as learning the standardized guideline for neonatal stabilization, during audits, junior learners can be trained when and how to adapt care for individual infants.

### Lessons Learned From Auditing Neonatal Stabilization

Audits at our NICU allowed providers to identify and address various areas for improvement. Oftentimes, improvements should be made by addressing knowledge and skill retention. Retention of knowledge and skills in the context of neonatal stabilization has been reported in various studies (21, 22), and boosting knowledge and skills is recommended (23). During audits, knowledge is constantly boosted. Moreover, receiving feedback on clinical performance allows for continuous development of expertise (20) and may increase self-assurance, which was also reported to improve clinical skills (24). Furthermore, insight in what actually happens in the delivery room allows to identify non-compliance to guidelines and open discussions about best practice, such as harmonizing and improving care provided by providers.

### Safe Learning Environment

Successful audits require a safe learning environment. Important prerequisites for a safe learning environment include a blame-free, shame-free environment focusing on the benefits for learning and improving, proper information about the goal of audits, involvement of providers, and secured storage of recordings (9).

In our experience, creating a safe learning environment is an ongoing process. This requires a lot of involvement of staff members and a process of auditing that is reshaped according to providers' needs. For our NICU, this meant that over time, audits evolved from meetings, in which stabilizations were anonymously discussed using standardized evaluation criteria (8), into open discussions, discussing clinical decision making and the appropriateness of interventions for individual patients, and boosting knowledge about, e.g., medical devices and standard operating procedures of delivery room studies. Providers who were involved in the stabilization are present, ask for feedback on their care, and add valuable information that may improve discussions.

During audits, staff members suggested further adaptations to improve the process of auditing. As such, providers that were involved in the stabilization are now informed beforehand and offered the opportunity to watch the recordings before the audit, or to prepare the audit together with the coordinator. Furthermore, stabilizations are only discussed when at least one of the involved providers can be present during the audit to provide extra information about the stabilization. This allows for more open discussions about the best care for the infant in the specific situation. Last, stabilizations are preferably not discussed the morning after a nightshift of one of the involved providers, as tiredness due to a (stressful) night shift may influence how feedback is received.

### Opportunities for Further Improvements

Auditing care provided during neonatal transition allows providers of our NICU to identify areas for improvement. These areas for improvement can then be addressed in Plan-Do-Study-Act (PDSA) cycles. PDSA cycles are widely used to drive quality improvement in healthcare and consist of four stages in which a change aimed at improvement is identified. This change is tested, the success of this change is studied, and consequently this change is implemented or adapted, starting a new PDSA cycle (25). During audits, processes that need to be improved are identified, and oftentimes, changes aimed at improvement are also discussed. By following the next stages of the PDSA cycle, we can systematically study the impact of these changes aimed at improvement. Doing so will not only be beneficial for our practice but also will improve the evidence base for auditing neonatal stabilization by providing outcome data about potential benefit for infants.

Analyzing captured lessons learned showed that lessons learned are beneficial for various disciplines, including nursing staff and obstetrics. However, audits are mostly attended by the medical team of the NICU. As such, lots of opportunities to improve care needlessly get lost. Future efforts should concentrate on organizing multidisciplinary meetings and studying the impact of such audits on improving patient safety and the quality of delivered care.

Successful neonatal stabilization demands a combination of technical and non-technical skills (4). In our practice, both technical and non-technical lessons learned were captured, yet the majority of lessons learned involved technical skills. This is probably related to the fact that in order to protect the privacy of our providers, our recordings do not include audio and only capture the infant and providers' hands. However, providers, themselves, preferred to openly discuss stabilizations, instead of anonymously. We hope to add audio to the recordings in the near future, so we can start auditing communication, teamwork, clinical decision making during stabilization, and other non-technical skills more in depthly.

## Conclusion

At our NICU, auditing neonatal stabilization improved guideline compliance and documentation in the medical record and is considered as educational and beneficial for improving the quality of care provided during neonatal stabilization. Furthermore, many lessons learned could be captured, and areas for improvement could be identified and addressed by boosting knowledge and skills. Most important identified areas for improvement were the usage of medical devices and the conduct of delivery room studies. Moreover, clinical decision making could be discussed plenarily, allowing the local guideline for neonatal stabilization to be clarified and improved. As such, audits contribute to improving patient safety and the quality of care provided during neonatal stabilization. By reporting our experiences, we hope to encourage other NICUs to also implement regular audit meetings, fitting to their improvement needs.

## Data Availability Statement

The raw data supporting the conclusions of this article will be made available by the authors, without undue reservation.

## Author Contributions

MB, TM, and MH drafted the initial version of the manuscript. All authors participated in critical revision of the manuscript for important intellectual content, approved the final manuscript as submitted, and agree to be accountable for all aspects of the work.

## Conflict of Interest

The authors declare that the research was conducted in the absence of any commercial or financial relationships that could be construed as a potential conflict of interest.

## References

[1] Schilleman K Siew ML Lopriore E Morley CJ Walther FJ te Pas AB. Auditing resuscitation of preterm infants at birth by recording video and physiological parameters. Resuscitation. (2012) 83:1135–9. 10.1016/j.resuscitation.2012.01.036 22322286

[2] Schilleman K Witlox RS van Vonderen JJ Roegholt E Walther FJ te Pas AB. Auditing documentation on delivery room management using video and physiological recordings. Arch Dis Childhood Fetal Neonatal Ed. (2014) 99:F485–90. 10.1136/archdischild-2014-306261 25125582

[3] Lloyd A Dewar A Edgar S Caesar D Gowens P Clegg G. How to implement live video recording in the clinical environment: a practical guide for clinical services. Int J Clin Pract. (2017) 71:e12951. 10.1111/ijcp.12951 28524616

[4] Yamada NK Kamlin COF Halamek LP. Optimal human and system performance during neonatal resuscitation. Semin Fetal Neonatal Med. (2018) 23:306–11. 10.1016/j.siny.2018.03.006 29571705

[5] Sawyer T Sierocka-Castaneda A Chan D Berg B Lustik M Thompson M. The effectiveness of video-assisted debriefing versus oral debriefing alone at improving neonatal resuscitation performance: a randomized trial. Simul Healthc. (2012) 7:213–21. 10.1097/SIH.0b013e3182578eae 22673159

[6] Nadler I Sanderson PM Van Dyken CR Davis PG Liley HG. Presenting video recordings of newborn resuscitations in debriefings for teamwork training. BMJ Qual Saf. (2011) 20:163–9. 10.1136/bmjqs.2010.043547 21216792

[7] Ades A Lee HC. Update on simulation for the neonatal resuscitation program. Semin Perinatol. (2016) 40:447–54. 10.1053/j.semperi.2016.08.005 27823817

[8] Root L van Zanten HA den Boer MC Foglia EE Witlox RSGM te Pas AB. Improving Guideline Compliance and Documentation Through Auditing Neonatal Resuscitation. Front Pediatr. (2019) 7:294. 10.3389/fped.2019.00294 31380327 PMC6646726

[9] den Boer MC Houtlosser M Foglia EE Tan RNGB Engberts DP te Pas AB. Benefits of recording and reviewing neonatal resuscitation: the providers' perspective. Arch Dis Child Fetal Neonatal Ed. (2018) 104:F528–F34. 10.1136/archdischild-2018-315648 30504441

[10] Kuypers K Lamberska T Martherus T Dekker J Bohringer S Hooper SB. The effect of a face mask for respiratory support on breathing in preterm infants at birth. Resuscitation. (2019) 144:178–84. 10.1016/j.resuscitation.2019.08.043 31521774

[11] Perlman JM Wyllie J Kattwinkel J Wyckoff MH Aziz K Guinsburg R. Part 7: neonatal resuscitation: 2015. International consensus on cardiopulmonary resuscitation and emergency cardiovascular care science with treatment recommendations (reprint). Pediatrics. (2015) 136(Suppl. 2):S120–66. 10.1542/peds.2015-3373D 26471381

[12] Wyllie J Bruinenberg J Roehr CC Rudiger M Trevisanuto D Urlesberger B. European resuscitation council guidelines for resuscitation 2015: section 7. Resuscitation and support of transition of babies at birth. Resuscitation. (2015) 95:249–63. 10.1016/j.resuscitation.2015.07.029 26477415

[13] Roehr CC O'Shea JE Dawson JA Wyllie JP. Devices used for stabilisation of newborn infants at birth. Arch Dis Child Fetel Neonatal Ed. (2018) 103:F66–71. 10.1136/archdischild-2016-310797 29079652

[14] Thomas AN Galvin I. Patient safety incidents associated with equipment in critical care: a review of reports to the UK national patient safety agency. Anaesthesia. (2008) 63:1193–7. 10.1111/j.1365-2044.2008.05607.x 18803628

[15] Mattox E. Medical devices and patient safety. Crit Care Nurse. (2012) 32:60–8. 10.4037/ccn2012925 22855080

[16] Sweetman EA Doig GS. Failure to report protocol violations in clinical trials: a threat to internal validity? Trials. (2011) 12:214. 10.1186/1745-6215-12-214 21955551 PMC3192675

[17] Ghooi RB Bhosale N Wadhwani R Divate P Divate U. Assessment and classification of protocol deviations. Perspect Clin Res. (2016) 7:132–6. 10.4103/2229-3485.184817 27453830 PMC4936072

[18] Mohan S Mehra M Petrizzo M Katz T. A toolkit for the management of protocol deviations. Ther Innov Regul Sci. (2016) 50:791–800. 10.1177/2168479016647987 30231739

[19] Carthey J Walker S Deelchand V Vincent C Griffiths WH. Breaking the rules: understanding non-compliance with policies and guidelines. BMJ. (2011) 343:d5283. 10.1136/bmj.d5283 21914756

[20] Liley HG Sanderson PM. More evidence for a “black box” to measure and improve outcomes in the delivery room. Resuscitation. (2018) 132:A3–4. 10.1016/j.resuscitation.2018.08.015 30138649

[21] Matterson HH Szyld D Green BR Howell HB Pusic MV Mally PV. Neonatal resuscitation experience curves: simulation based mastery learning booster sessions and skill decay patterns among pediatric residents. J Perinatal Med. (2018) 46:934–41. 10.1515/jpm-2017-0330 29451862

[22] Patel J Posencheg M Ades A. Proficiency and retention of neonatal resuscitation skills by pediatric residents. Pediatrics. (2012) 130:515–21. 10.1542/peds.2012-0149 22926169

[23] Sawyer T Ades A Ernst K Colby C. Simulation and the neonatal resuscitation program 7th edition curriculum. NeoReviews. (2016). 17:e447–53. 10.1542/neo.17-8-e44728096704

[24] Malmstrom B Nohlert E Ewald U Widarsson M. Simulation-based team training improved the self-assessed ability of physicians, nurses and midwives to perform neonatal resuscitation. Acta Paediatr. (2017) 106:1273–9. 10.1111/apa.13861 28370414

[25] Taylor MJ McNicholas C Nicolay C Darzi A Bell D Reed JE. Systematic review of the application of the plan-do-study-act method to improve quality in healthcare. BMJ Qual Saf. (2014) 23:290–8. 10.1136/bmjqs-2013-001862 24025320 PMC3963536

